# Dual-Energy Computed Tomography For Differentiation Between Osteoblastic Metastases and Bone Islands

**DOI:** 10.3389/fonc.2022.815955

**Published:** 2022-07-12

**Authors:** Chijie Xu, Lingling Kong, Xiaoyi Deng

**Affiliations:** Department of Radiology, Afliated AoYang Hospital of Jiangsu University, Zhangjiagang, China

**Keywords:** dual-energy computed tomography, osteoblastic metastasis, bone island, effective atomic number, electron density

## Abstract

**Objective:**

The objective of our study was to evaluate the utility of Rho/Z on dual-energy computed tomography (DECT) for the differentiation of osteoblastic metastases (OBMs) from bone islands (BIs).

**Methods:**

DECT images of 110 patients with malignancies were collected. The effective atomic number (Z), electron density (Rho), dual energy index (DEI), and regular CT (rCT) values were measured by two observers. Independent-sample *t*-test was used to compare these values between OBMs and BIs. The diagnostic performance was assessed by receiver operating characteristic (ROC) analysis and the cutoff values were evaluated according to ROC curves.

**Results:**

A total of 205 OBMs and 120 BIs were included. The mean values of Z, Rho, DEI, and rCT of OBMs were significantly lower than those of BIs, whereas the standard deviation values were higher than those of BIs (all *p* ≤ 0.05). ROC analysis showed that 11.86 was the optimal cutoff value for Z, rendering an area under the ROC curve (AUC) of 0.91, with a sensitivity of 91.2% and a specificity of 82.5%.

**Conclusion:**

DECT can provide quantitative values of Z, Rho, and DEI and has good performance in differentiating between OBMs and BIs.

## Introduction

With the increasing risks of bone metastases in patients with malignancies, clinicians need to detect bone metastasis at the early stage to reduce complications of bone metastasis. Osteoblastic metastases (OBMs) and bone islands (BIs) must be included in the differential diagnosis of malignancies and newly identified sclerotic osseous lesions. The differential diagnosis of OBMs and BIs is crucial because it has great significance for the staging, treatment, and prognosis of patients.

Conventional CT is essential to the diagnosis of metastasis and the detection of progressive metastasis. Thus, it is integral to the follow-up study of patients with malignancies. CT also provides important information for choosing appropriate treatments in clinical practice. However, due to the severe beam-hardening artifacts and difficulty in visually differentiating overlapping CT values on conventional CT images, it is still challenging to differentiate between OBMs and BIs.

Recently, DECT has begun to be widely used in musculoskeletal imaging (1–4). From DECT image data, the electron density (Rho), effective atomic number (Z), and dual energy index (DEI) can be obtained. These variables describe the composition of a scanned object; thus, they can be used to differentiate materials, especially materials with relatively high atomic numbers (e.g., iodine and bone). Compared to conventional CT, the advantage of DECT is that it enables material differentiation *via* analysis of the Compton and photoelectric effects without increasing the radiation dose. The accuracy of Rho/Z post-processing has been evaluated and reported in studies on thorax and abdomen oncologic imaging, radiotherapy, and uric acid stone analysis (5–7). Saito stated a single linear relationship between CT number and Rho/Z through the conversion from the energy-subtracted CT number by means of DECT to the relative electron density of the material (8). Bharati showed that DECT can be used to calculate the effective atomic number and electron density with good accuracy (9). The aim of this study was to evaluate the utility of Rho/Z on DECT for the differentiation of OBMs from BIs.

## Materials and Methods

### Patient Selection Protocol

This study was approved by the institutional review board of Aoyang hospital and written informed consent was waived owing to retrospective data. A retrospective review was performed for oncologic patients attending this hospital from March 2018 to October 2021. Inclusion criteria were as follows: (1) the primary malignant tumors of the patient were confirmed by pathological biopsy; (2) the patient did not receive anti-tumor treatment before DECT examinations; (3) the focal hyperdense intra-osseous lesions (ranging from 0.5 cm to 2.0 cm) were first found by DECT; and (4) MRI or PET-CT and follow-up CT imaging with a duration of longer than 6 months performed at this hospital were available for the diagnosis of lesions without pathological results. Exclusion criteria were as follows: (1) clinical or imaging data were incomplete; and (2) the diagnosis of hyperdense intra-osseous lesions was not clear.

The reference standard for BIs was pathologically proved or typical radiologic appearance: (a) hyperdense intra-osseous lesions without morphological features, (b) no radionuclide uptake on bone scintigraphs or enhanced after MRI enhancement, and (c) no change in the size and/or density on 6 months or longer follow-up CT images (10). The reference standard for OBMs was pathologically proved or typical radiologic appearance: (a) hyperdense intra-osseous lesions with a nodular appearance, (b) increased radionuclide uptake on bone scintigraphs or enhanced after MRI enhancement, (c) an increase in the size and/or density on 6 months or longer follow-up CT images, and (d) lack of compression fracture or pathologic fracture.

### DECT Imaging Protocol

All patients were positioned supine feet first on the scanning couch and underwent a non-contrast chest or abdominal CT scan using a DECT scanner (Somatom Definition Flash, Siemens Healthcare, Forchheim, Germany). The CT protocols were as follows: collimation 40 × 0.6 mm, pitch 1.2, rotation time 0.5 s, tube voltages of 100 kV (tube A) and 140 kV (tube B) with tube currents of 250 reference mAs (tube A) and 483 reference mAs (tube B), and automated attenuation-based tube current modulation (Care Dose 4D, Siemens).

### Quantitative Analysis

**Figure 1:**
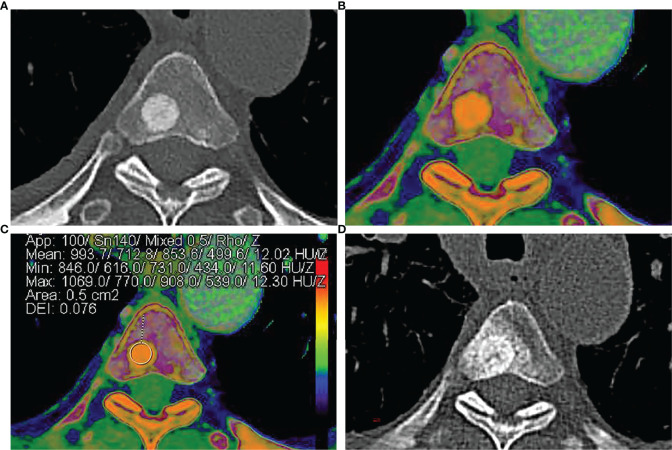
A 58-year-old man was diagnosed with lung adenocarcinoma. **(A)** Axial contrast-enhanced CT image shows an intra-osseous focal hyperdense lesion with a clear margin in the 4th thoracic vertebral body. **(B, C)** Rho/Z on DECT shows the placement of ROI over an intra-osseous focal hyperdense lesion. Values of ROI: Z = 12.02, Rho = 499.6, DEI = 0.076, rCT = 853.6. **(D)** Size and density of the intra-osseous focal hyperdense lesion were both increased in 1-year follow-up CT images, and the lesion was diagnosed as OBM.

**Figure 2:**
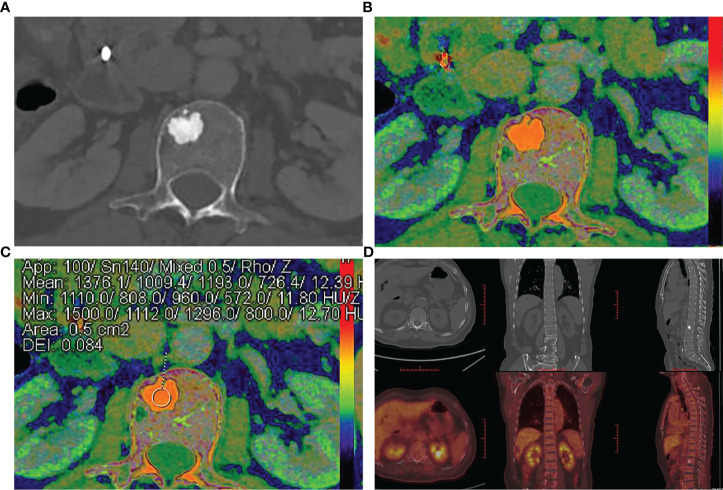
A 70-year-old man was diagnosed with cholangiocarcinoma. **(A)** Axial contrast-enhanced CT image shows an intra-osseous focal hyperdense lesion with a clear margin in the 2nd lumbar vertebral body. **(B, C)** Rho/Z on DECT shows the placement of ROI over an intra-osseous focal hyperdense lesion. Values of ROI: Z = 12.39, Rho = 726.4, DEI = 0.084, rCT = 1193.0 HU. **(D)** Intra-osseous focal hyperdense lesion showed no increased radionuclide uptake on bone scintigraphs, and it was diagnosed as BI.

Two radiologists (with 8–10 years of experience in orthopedic imaging) performed quantitative analysis. The values of Z, Rho, and DEI were measured using a dedicated software for DECT in our workstation (Rho/Z, Syngo.via, Version VB20, Siemens Healthineers). The radiologists were blinded to the final diagnosis, follow-up imaging, and medical history. For each lesion, oval or circular ROIs were placed on DECT images (**Figures 1**, **2**). The ROI was placed along the lesion to fit as much of the lesion as possible and measured the maximum dimension avoiding the bone cortex. The ROI area should not to be less than 0.5 cm^2^.

### Statistical Analysis

Statistical analysis was performed using SPSS 19.0. All continuous variables were expressed as mean ± standard deviation (SD). Inter-reader agreement for measurements on DECT was evaluated by *κ* statistics. Independent-sample *t*-test was used to compare patients’ gender, age, follow-up time, ROI area, and the measurements of Z, Rho, DEI, and rCT values between OBMs and BIs. The differences between OBM subtypes (lung cancer group, breast cancer group, prostate cancer group, gastric cancer group, and rectal cancer group) and BIs were analyzed by one-way ANOVA. Receiver operating characteristic (ROC) curve analysis was performed to compare the area under the curve (AUC) for the differential diagnostic efficacy of OBMs from BIs using parameter measurements. The optimal cutoff values for this differential diagnosis were determined and evaluated in terms of their sensitivity and specificity. A *p*-value of less than 0.05 was regarded as statistically significant.

## Results

### Patient Characteristics

This study included 110 patients with a total of 325 lesions. Among them, 31 patients (mean age, 64.2 ± 9.2 years; age range, 42–78 years; 17 men and 14 women) with 205 OBMs and 79 patients (mean age, 66.6 ± 9.7 years; age range, 33–81 years; 51 men and 28 women) with 120 BIs were determined. There was no significant difference in patients’ gender (*p* = 0.35), age (*p* = 0.23), or follow-up time (*p* = 0.08) between OBMs and BIs. The mean ROI area of OBMs was 0.68 ± 0.08 cm^2^, and the mean ROI area of BIs was 0.67 ± 0.11 cm^2^. There was no significant difference in ROI area (*p* = 0.08) between OBMs and BIs. The inter-observer agreement was excellent (*κ* = 0.83). Clinical and demographic data are shown in **Table 1**.


$${\text{Z}}_{\text{mean}},{\text{ Rho}}_{\text{mean}},{\text{ DEI}}_{\text{mean}},{\text{ and rCT}}_{\text{mean}}\text{ values}$$

**Table 1:** Patient characteristics.

	OBMs		BIs	
Gender (male/female)	17/14		51/28	
Age (years)	66		68	
Follow-up time (months)	9.3 ± 2.8		10.8 ± 4.8	
ROI area (cm^2^)	0.68 ± 0.08		0.67 ± 0.11	
Lesions	205		120	
Primary tumor type (%)	No. of patients	No. of lesions	No. of patients	No. of lesions
Lung cancer	11 (35.4%)	90 (43.9%)	13 (16.5%)	19 (15.8%)
Breast cancer	7 (22.5%)	60 (29.3%)	7 (9.0%)	11 (9.2%)
Prostate cancer	6 (19.3%)	30 (14.6%)	1 (1.3%)	1 (0.8%)
Rectal cancer	2 (6.0%)	5 (2.4%)	8 (10.0%)	11 (9.2%)
Gastric cancer	2 (6.0%)	11 (5.4%)	17 (21.5%)	25 (20.8%)
Ovarian cancer	1 (3.0%)	6 (2.9%)	0	0
Nasopharyngeal cancer	1 (3.0%)	2 (0.9%)	0	0
Cholangiocarcinoma	1 (3.0%)	1 (0.5%)	1 (1.3%)	1 (0.8%)
Colon cancer	0	0	18 (22.8%)	27 (22.5%)
Esophageal cancer	0	0	5 (6.3%)	8(6.7%)
Pulmonary synovial sarcoma	0	0	1 (1.3%)	1 (0.8%)
Thymus cancer	0	0	1 (1.3%)	4 (3.3%)
Gallbladder cancer	0	0	1 (1.3%)	1 (0.8%)
Kidney cancer	0	0	2 (2.5%)	2 (1.7%)
Osteosarcoma	0	0	1 (1.3%)	1 (0.8%)
Melanoma	0	0	1 (1.3%)	1 (0.8%)
Pancreatic cancer	0	0	1 (1.3%)	3 (2.5%)
Duodenal cancer	0	0	1 (1.3%)	1 (0.8%)

**Figure 3:**
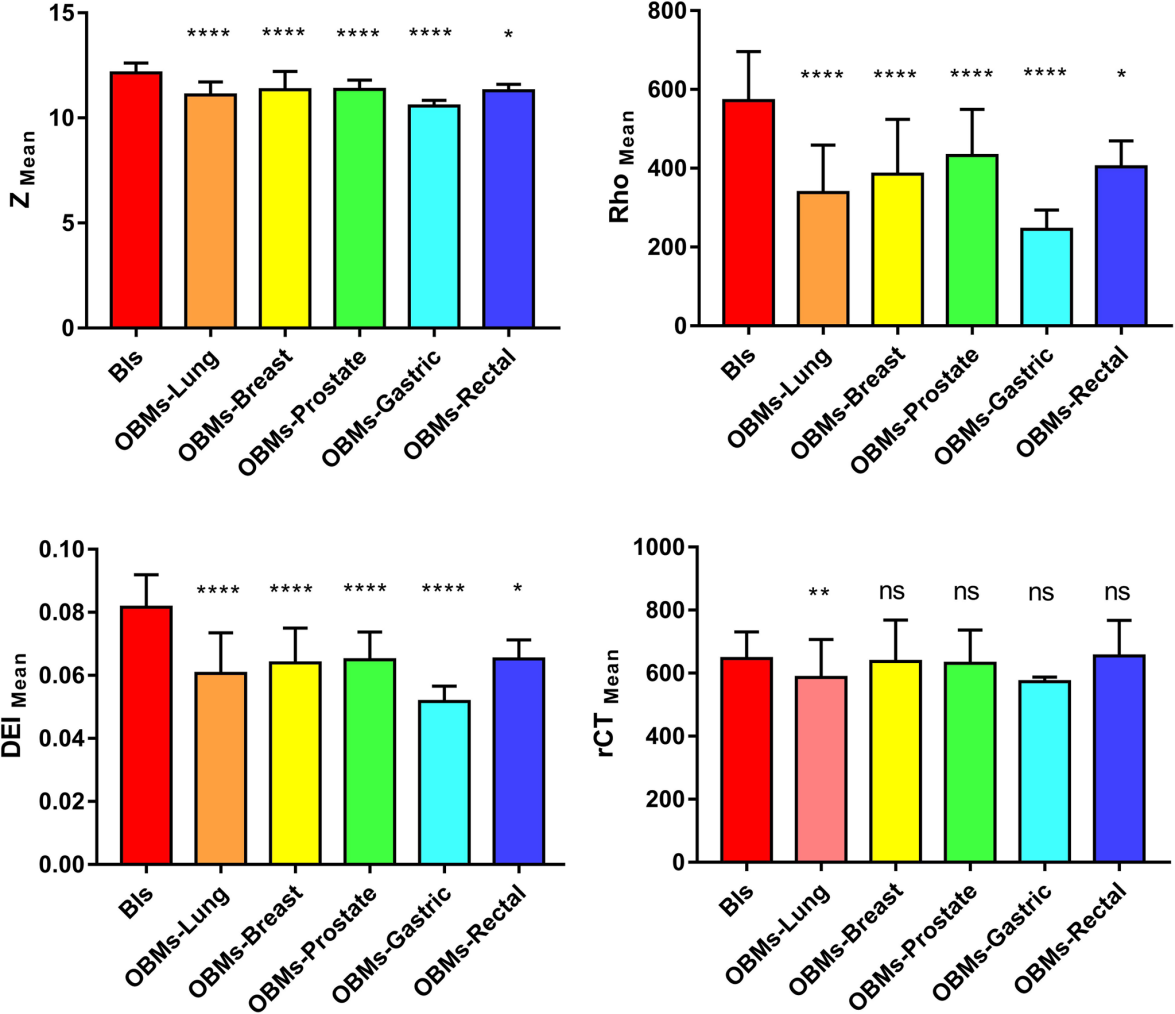
Note. *p < 0.05, **p < 0.01, ****p < 0.001, ns, not significant. Box-whisker plots of Zmean, Rho_mean_, DEI_mean_, and rCT_mean_ values for BIs and OBM-subtypes.

The Z_mean_ values of BIs and OBMs were 12.14 ± 0.48 and 11.18 ± 0.68, respectively. The Rho_mean_ values for BIs and OBMs were 571.57 ± 124.56 and 359.81 ± 128.92, respectively. The DEI_mean_ values for BIs and OBMs were 0.08 ± 0.01 and 0.06 ± 0.01, respectively. The rCT_mean_ values for BIs and OBMs were 646.04 ± 85.36 and 606.78 ± 119.19, respectively. The Z_mean_, Rho_mean_, DEI_mean_, and rCT_mean_ values of BIs were higher than those of OBMs. There was no statistically difference in rCT_mean_ values between BIs and OBMs (*p* = 0.002), whereas there were statistically significant differences in Z_mean_, Rho_mean_, and DEI_mean_ values between BIs and OBMs (*p* < 0.001). In the subtype analyses, there was significant difference in the rCT_mean_ values between lung cancer OBMs and BIs (*p =* 0.001), while there were no significant differences in the rCT_mean_ values between other groups and BIs (all *p* > 0.05). There were significant differences in Z_mean_, Rho_mean_, and DEI_mean_ values on DECT between all subgroups and BIs (all *p* ≤ 0.05) (**Table 2**, **Figure 3**).

**Table 2:** Z_mean_, Rho_mean_, DEI_mean_, and rCT_mean_ values of DECT for BIs and OBMs.

	BIs (*n* = 120)	OBM-subtypes											
		Total(*n* = 205)	*p-*value	Lung cancer(*n* = 90)	*p*-value	Breast cancer(*n* = 60)	*p*-value	Prostate cancer(*n* = 30)	*p*-value	Gastric cancer(*n* = 11)	*p*-value	Rectal cancer(*n* = 5)	*p-*value
Z	12.14 ± 0.48	11.18 ± 0.68	<0.001	11.18 ± 0.58	<0.001	11.43 ± 0.94	<0.001	11.40 ± 0.41	<0.001	10.57 ± 0.26	<0.001	11.33 ± 0.27	0.03
Rho	571.57 ± 124.56	359.81 ± 128.92	<0.001	354.89 ± 118.01	<0.001	401.69 ± 135.60	<0.001	442.95 ± 108.89	<0.001	244.98 ± 47.02	<0.001	411.17 ± 57.40	0.04
DEI	0.08 ± 0.01	0.06 ± 0.01	<0.001	0.06 ± 0.01	<0.001	0.07 ± 0.01	<0.001	0.07 ± 0.01	<0.001	0.05 ± 0.01	<0.001	0.07 ± 0.01	0.01
rCT	646.04 ± 85.36	606.78 ± 119.19	0.002	610.77 ± 125.95	0.001	663.78 ± 140.71	0.995	648.35 ± 110.06	0.984	573.45 ± 13.74	0.261	670.16 ± 100.97	0.998

### Diagnostic Performance for Differentiation Between BIs and OBMs

**Figure 4:**
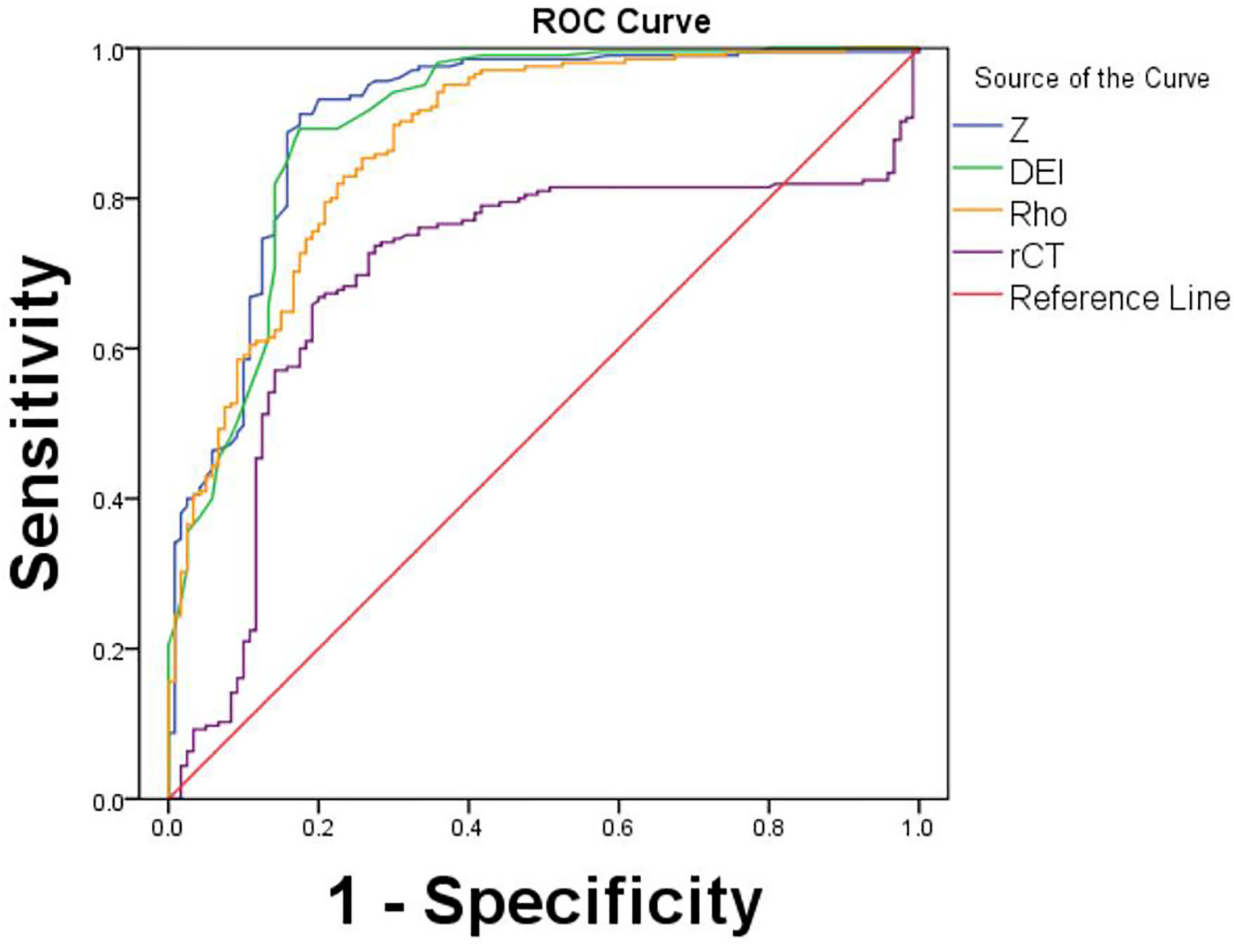
ROC curves for Z, Rho, DEI, and rCT values by the Rho/Z on DECT for differentiation between OBMs and BIs.

The AUCs for Z_mean_ (AUC = 0.91), Rho_mean_ (AUC = 0.88), and DEI_mean_ (AUC = 0.90) were higher than that for rCT_mean_ (AUC = 0.70). ROC curve analysis showed that Z_mean_ had the highest AUC value and the maximal diagnostic performance in differentiating OBMs from BIs. The optimal cutoff value for Z_mean_ was 11.86, which had a sensitivity of 91.2% and a specificity of 82.5% (**Table 3**, **Figure 4**).

**Table 3:** AUC values, optimal cutoff values, sensitivity, and specificity for differentiation between BIs and OBMs.

	AUC	Cutoff values	Sensitivity (%)	Specificity (%)	95% CI
Z	0.91	11.86	91.20	82.50	86.90–94.20
Rho	0.88	536.85	89.80	70.00	83.50–91.40
DEI	0.90	0.08	89.30	82.50	86.10–93.70
rCT	0.70	618.10	66.80	80.00	63.50–75.80

## Discussion

According to our study, OBMs can be differentiated from BIs by the Rho/Z on DECT. The Z, Rho, and DEI values for BIs were found to be significantly higher than those for OBMs in this study. These quantitative parameters on DECT show statistically significant differences with rCT values. We achieved higher sensitivity and specificity for differentiation between OBMs and BIs by using multi-parameter quantitative values on DECT images. The optimal cutoff value of 11.86 for Z had an AUC of 0.91, with 91.20% sensitivity and 82.50% specificity.

CT values are presented as the mean of the CT values of all the individual pixels included in the ROI. A recent study showed that CT’s sensitivity and specificity for bone metastasis were 75.6% and 89.2%, respectively (11). Another study by Ulano et al. mentioned the usage of CT attenuation on conventional CT scanners to differentiate BIs from OBMs (12). They found that a threshold mean CT attenuation of 885 HU had an AUC of 0.982, and a threshold maximum CT attenuation of 1060 HU had an AUC of 0.976. Study results reported by Elangovan et al. and Sala et al. were similar to the findings reported by Ulano et al. (13, 14). The sensitivity and specificity of rCT values in our study (66.80% and 80.00%, respectively) were lower. As the distribution of lesions may have affected the threshold, only the spinal and pelvis lesions were evaluated in our study, which could be the reason for the higher sensitivity in their study. Different types of primary tumors may affect the threshold; thus, we performed five subtype analyses for comparison between OBMs and BIs. Compared to conventional CT, our results showed that DECT multiple quantitative parameters can better help differentiate between OBMs from different types of primary tumors and BIs. In addition, since OBMs are common in elderly patients with malignancies, age- and gender-matched controls may be better in our study. However, recent studies on rCT value analysis of metastatic lesions were sparse and mostly contradictory due to the overlapping CT values and beam hardening artifacts on conventional CT images, which bias the results.

DECT can improve lesion depiction, image quality, and material differentiation by separately administering low- and high-energy photons to obtain images such as virtual monoenergetic images and virtual noncontrast (VNC) images. DECT can be divided into detector- and tube-based approaches. Currently, four DECT methods are used in clinical practice: (1) dual tubes with or without beam filtration, (2) rapid voltage switching with single tube, (3) dual-layer detector with single tube, and (4) single tube with split filter or sequential dual scans. Recently, a new post-processing approach of Rho/Z on DECT was proposed for differentiating OBMs and BIs. It can provide multi-parameter quantitative analysis by determining the electron density (Rho), effective atomic number (Z), and dual energy index (DEI) values, which can help reduce beam hardening artifacts and make more accurate assessments (15). Rho represents the number of electrons per unit volume and has a linear relationship with the CT values. Z represents the atomic number of an element and is more sensitive to materials with relatively high atomic numbers (e.g., iodine and bone), exhibiting large differences at different energy levels (16–18). DEI is calculated from the relative difference in the attenuation values of a material at different energy photon energies, suggesting that it is an indicator with reliability and effectiveness for differentiating various types of materials (19). Saito et al. showed that CT value is related to the electron density, atomic number, and DEI of materials and has a linear relationship with each of these variables (8), but we proved that the Rho/Z on DECT has a better performance in the differentiation between OBMs and BIs. The increased sensitivity and specificity of DECT can obviate the need for additional imaging.

In addition, the SD value suggests the degree of divergence of the individual pixel values from the mean value, reflecting the homogeneity within the tissue. A higher SD value indicates less homogeneity within the tissue. BIs are mature cortical bones that grow along cancellous bone and contain cancellous bone trabeculae, normal bone marrow, and cortical bone. OBMs are more heterogeneous, since the normal bone tissue is replaced by various types of tumor cells. Our study shows that OBMs have lower density and higher SD value compared to BIs, which agrees with the study by Dong et al. (20). Nevertheless, MRI is still the method most frequently used to diagnose bone metastasis. The Rho/Z on DECT is not appropriate as a substitute for MRI, although it may serve as an auxiliary tool for differentiation between OBMs and BIs, and it could be useful for patients with contraindications to MRI. It is necessary to explore the correlation of multi-parameter quantitative values obtained by DECT with other modalities, such as MRI, ECT, or PET-CT. More studies need to be performed in the future to explore the value of DECT multi-parameter imaging for differentiation between OBMs and BIs.

Our study has some limitations. First, this study is retrospective, with possible selection bias. This study also has a small sample size; thus, the results still need to be further confirmed by large-sample, multi-center studies in the future. Second, not all lesions were diagnosed with pathologic examination. Most of the lesions were diagnosed with typical radiologic findings (MRI, ECT, or PET-CT) and follow-up imaging. Third, the parameter threshold of patients with different pathological types and different tumor metabolism needs to be further studied.

In conclusion, DECT can be used for quantitative analysis of material composition by providing multiple quantitative parameters, namely, Z, Rho, and DEI. We determined the optimal cutoff value for Z and demonstrated its sufficient potential for differentiating between OBMs and BIs, thereby contributing to more accurate diagnosis.

## Data Availability Statement

The raw data supporting the conclusions of this article will be made available by the authors, without undue reservation.

## Ethics Statement

The studies involving human participants were reviewed and approved by Ethics Committee of Zhangjiagang Aoyang Hospital. The patients/participants provided their written informed consent to participate in this study.

## Author Contributions

CX was responsible for the article writing; DX (corresponding author) was responsible for the article guidance; LK was co-first author and had contributed equally to this work. All authors contributed to the article and approved the submitted version.

## Conflict of Interest

The authors declare that the research was conducted in the absence of any commercial or financial relationships that could be construed as a potential conflict of interest.

## Publisher’s Note

All claims expressed in this article are solely those of the authors and do not necessarily represent those of their affiliated organizations, or those of the publisher, the editors and the reviewers. Any product that may be evaluated in this article, or claim that may be made by its manufacturer, is not guaranteed or endorsed by the publisher.

## Abbreviations

OBMs, Osteoblastic metastases; BIs, Bone islands; MRI, Magnetic resonance imaging; ECT, Emission computed tomography; DECT, Dual-energy computed tomography; PET, Positron emission tomography; Rho, Electron density; Z, Effective atomic number; DEI, Dual energy index; rCT, Regular CT value; ROI, Region of interest; ROC, Receiver operating characteristic; AUC, Area under the ROC curve.
